# Involvement of Heme in Colony Spreading of *Staphylococcus aureus*

**DOI:** 10.3389/fmicb.2020.00170

**Published:** 2020-02-11

**Authors:** Chao-Chin Liu, Mei-Hui Lin

**Affiliations:** ^1^Graduate Institute of Biomedical Sciences, College of Medicine, Chang Gung University, Taoyuan, Taiwan; ^2^Department of Medical Biotechnology and Laboratory Science, College of Medicine, Chang Gung University, Taoyuan, Taiwan; ^3^Department of Laboratory Medicine, Chang Gung Memorial Hospital, Taoyuan, Taiwan

**Keywords:** *Staphylococcus aureus*, colony spreading, water accumulation, heme, ATP

## Abstract

*Staphylococcus aureus* spreads rapidly on the surface of soft agar medium. The spreading depends on the synthesis of biosurfactants, i.e., phenol soluble modulins (PSMs), which facilitate colony spreading of *S. aureus*. Our earlier study demonstrated that water accumulates in a colony is important to modulate colony spreading of *S. aureus*. The current study screened a transposon-based mutant library of *S. aureus* HG001 and obtained four non-spreading mutants with mutations in *hemY* and *ctaA*, which are involved in heme synthesis. The spreading ability of these mutants was restored when the mutants are transformed with a plasmid encoding *hemY* or *ctaA*, respectively. *HemY* mutants, which do not synthesize heme B, were able to spread on agar medium supplemented with hemin, a heme B derivative. By contrast, hemin supplementation did not rescue the spreading of the *ctaA* mutant, which lacks heme B and heme A, indicating that heme A is also critical for colony spreading. Moreover, mutations in *hemY* and *ctaA* had little effect on PSMs production but affect ATP production and water accumulation in the colony. In conclusion, this study sheds light on the role of heme synthesis and energy production in the regulation of *S. aureus* colony spreading, which is important for understanding the movement mechanisms of bacteria lacking a motor apparatus.

## Introduction

Bacteria use different types of motility, including swarming, swimming, twitching, gliding, and sliding, to move across a surface or through a liquid to avoid dangers and acquire nutrients (Olsen et al., 2013; Vater et al., 2014). Many pathogenic members of the genera *Proteus*, *Serratia*, *Vibrio*, *Bacillus*, *Clostridium*, *Escherichia*, and *Salmonella* swarm, which is critical to the establishment of infection by these bacteria (Allison et al., 1992; Jones et al., 2004; Kim and Surette, 2005; Lai et al., 2005; Callegan et al., 2006; Jaques and McCarter, 2006; Armbruster et al., 2013). However, *Staphylococcus aureus*, which does not have pili or flagella, uses a different strategy, called colony spreading, to move across the surface of soft agar and animal tissues (Kaito and Sekimizu, 2007).

Many factors, including wall teichoic acid (WTA) and extracellular DNA, are involved in colony spreading of *S. aureus* (Kaito and Sekimizu, 2007; Kaito et al., 2011a). Furthermore, cell wall associated factors such as fibronectin-binding protein A/B (FnbpA/B) and clumping factor A/B (ClfA/B), both of which promote biofilm formation (O’Neill et al., 2008), antagonize colony spreading (Tsompanidou et al., 2012). Recent studies have demonstrated that host factors, such as albumin and high-density lipoproteins in serum, stimulate *S. aureus* colony spreading (Omae et al., 2014). Additionally, spreading motility depends on the activation of the accessory gene regulator (Agr) quorum-sensing system, which is responsible for the expression of the biosurfactant phenol-soluble modulins (PSMs) (Tsompanidou et al., 2013). *S. aureus* expresses a variety of PSMs, including PSMα1–4, PSMβ1–2, and PSMγ, with PSMγ more commonly known as δ-hemolysin (Queck et al., 2008; Aoyagi et al., 2014). Among the PSMs produced by *S. aureus*, PSMα1–4 are the most important for colony spreading (Tsompanidou et al., 2013). By contrast, colony spreading of *S. aureus* is inhibited by PSMγ (Omae et al., 2012) and the newly identified *psm-mec* gene; *psm-mec* is located in the *fudoh* locus in the type II and III staphylococcal chromosomal cassettes *mec* (SCC*mec*) (Kaito et al., 2008), which also contains the *mecA* gene responsible for methicillin resistance (Kaito et al., 2011b).

A recent study on *Bacillus subtilis* swarming revealed that the organism extracts water from agar and produces surfactin, which reduces the surface tension of water (Ke et al., 2015), ultimately allowing the colony to form flowing water-filled channels that facilitate the swarming of bacteria, resulting in rapid expansion of the colony (Ke et al., 2015). Extraction of water from agar to facilitate swarming is not limited to *B. subtilis*. Water is also found in swarms formed by *Escherichia coli* and *Salmonella enterica* (Chen et al., 2007; Ping et al., 2014). Our earlier study found that water accumulates in spreading colonies of *S. aureus* (Lin et al., 2016). In *E. coli*, it has been suggested that lipopolysaccharide (LPS) serves as an osmolyte to extract water from agar (Ping et al., 2014). However, *B. subtilis* and *S. aureus* do not contain LPS, and whether these organisms use an osmolyte to extract water remains unknown. To further investigate the mechanisms of colony spreading, this study screened a transposon-based mutant library of *S. aureus* HG001 and obtained non-spreading mutants with mutations in genes involved in the synthesis of heme, an iron-containing porphyrin compound that participates in aerobic respiration and energy production (Hederstedt, 2012; Dailey et al., 2017). The results demonstrate that heme deficiency has little effect on PSMs expression but greatly impacts ATP production. Moreover, the spreading colonies of heme-deficient mutants accumulate less water, indicating that heme plays a role in energy generation and water extraction during colony spreading.

## Materials and Methods

### Strains, Culture Conditions, and Chemicals

*Staphylococcus aureus* HG001, a derivative of *S. aureus* NCTC8325 (Herbert et al., 2010), was used in a spreading assay and for the generation of a transposon mutant library. M47, M99, D19, and M60 mutants with defects in colony spreading were selected from the transposon mutant library using a spreading assay (Table 1). *E. coli* EPI300 (Epicenter Technologies, Madison, WI, United States) was used as a host for cloning. *S. aureus* RN4220, which produces β-hemolysin (Nair et al., 2011), were used for assessment of δ-hemolytic activity. *S. aureus* SA113 (ATCC35556) and its isogenic mutant SA113Δ*tagO*, which contains a deletion in *tagO* and does not produce WTA (Weidenmaier et al., 2004) were used in a spreading assay, tiled plate assay, and ATP assay. *S. aureus* NCTC8325-4, a derivative of *S. aureus* NCTC8325 and its isogenic mutant NCTC8325-4Δ*hemB*, which contains a deletion in *hemB* and does not produce hemes (von Eiff et al., 1997), were used in a spreading assay, tiled plate assay, ATP assay, and enzyme activity assay. Bacteria were cultured in tryptic soy broth (TSB) and tryptic soy agar (TSA) (Oxoid, Basingstoke, United Kingdom). Antibiotic-resistant colonies were selected on media that contained ampicillin (100 μg/ml), spectinomycin (100 μg/ml), erythromycin (5 μg/ml), and chloramphenicol (10 μg/ml). Hemin, tunicamycin and *N,N′*-dicyclohexylcarbodiimide (DCCD) purchased from Sigma–Aldrich, Inc. (St. Louis, MO, United States) were added to TSA-0.25 and TSA-0.4.

**TABLE 1:** Spreading mutants of *S. aureus* HG001.

Mutant	Gene	Protein	Insertion site (n.t.)^a^	Function
M47	*hemY*	Protoporphyrinogen oxidase	773	Heme B synthesis
M60	*ctaA*	Heme A synthase	1023	Heme A synthesis
M99	*hemY*	Protoporphyrinogen oxidase	937	Heme B synthesis
D19	*hemY*	Protoporphyrinogen oxidase	150	Heme B synthesis
M253	*agrC*	AgrC	287	Agr quorum-sensing system
M480	*agrC*	AgrC	647	Agr quorum-sensing system
CGL005	*agrD*	AgrD	74	Agr quorum-sensing system

*^*a*^Transposon insertion site; nucleotide (n.t.) number from translation initiation codon.*

### Spreading and Gravity-Flow Experiments

For spreading assays, TSA containing 0.25 (TSA-0.25) or 0.4% (TSA-0.4) agarose (Seakem, Lonza Rockland, Inc.) was used. After autoclaving, medium was placed in a water bath at 55°C for 30 min. Plates containing 25 ml TSA-0.25 or TSA-0.4 were prepared and were then incubated at room temperature for 20 min before inoculation. Each plate was inoculated with 2 μl (1 × 10^7^ CFU) of an overnight culture and was then allowed to dry for 15 min in a laminar flow hood. The plates were incubated at 37°C for 24 h to allow bacteria to spread. To determine the presence of water within colonies, the TSA-0.4 plates were tilted at a 30° angle during incubation according to the method described previously (Lin et al., 2016).

### Transposon Mutagenesis

A mutant library was generated using the *bursa aurealis* transposon-based insertional mutagenesis method (Bae et al., 2004). Briefly, *S. aureus* HG001 was sequentially transformed with pBursa and pFA545, which contains genes encoding mariner transposase and confers resistance to tetracycline and ampicillin (Bae et al., 2004). The resulting transformants were spread on TSA containing erythromycin (TSAerm) and were incubated at 30°C overnight. After induction of transposase expression and curing the plasmid by incubating the transformants at 43°C for 2 days, cells containing transposon insertions in the chromosome were selected on TSAerm. Approximately 9000 mutants were collected and screened for their inability to spread on TSA-0.25 plates. Mutants that were defective in spreading were identified using a spreading assay (Lin et al., 2016). The sites of transposon insertion in the selected mutants were analyzed by sequencing as previously described (Bae et al., 2004). Briefly, total DNA of *S. aureus* was isolated and digested with the restriction enzyme *Aci*I. The digested DNA fragments were self-ligated and amplified by inverse PCR using primers complementary to the border sequences of the transposon (Bae et al., 2004). The PCR products were then sequenced to identify the location of transposon insertion using the genome sequence of *S. aureus* NCTC8325 (accession number: NC_007795.1) (Herbert et al., 2010), which is the parental strain of *S. aureus* HG001.

### Plasmids

The plasmid pPSM-*gfp* (Lin et al., 2016), which contains the *gfp* gene transcribed from the *psm*α promoter, was used to determine quorum-sensing activation in spreading colonies. The *hemY* gene was amplified by PCR using the primers hemY-F (5′-CGGTCTAGAGGTGGTTTATCACCATTAGC) and hemY-R (5′-TTACCCGGGCTTACAACTCTGCGATTACT); *ctaA* was amplified using the primers ctaA-F (5′-CGGTCTAGA GCGTGCCATTAAAATTACGG) and ctaA-R (5′-TTACCCGGG ATGCGCCACCCATAATTAAA) and *tagO* was amplified using the primers tagO-F (5′-CGGTCTAGATAGCACTTGTT ACTGCAGCA) and tagO-R (5′-TTACCCGGGATCCCATA CAGCTATGCTTT). The amplified DNA fragments were digested with *Xba*I and *Sma*I and were then inserted into the *Xba*I–*Sma*I sites in pHY300PLK (TaKaRa, Japan) to generate pHY-*hemY*, pHY-*ctaA*, and pHY-*tagO*.

### PSMs Extraction and Detection

Phenol soluble modulins were extracted according to a method described previously (Lin et al., 2018). Briefly, an overnight culture was harvested and adjusted with TSB to an OD_578_ of 7.0. Following centrifugation at 2000 × *g* for 10 min, the supernatants were separated into two parts. One part of supernatant was used to extract PSMs using a butanol method (Lin et al., 2018). The other part of supernatant was used to isolate the exoproteins using Amicon Ultra-4 centrifugal filters (Millipore, Billerica, MA, United States). Both PSMs and exoproteins were analyzed using SDS-PAGE, followed by Coomassie blue staining (0.1% Coomassie brilliant blue R250, 10% acetic acid, 50% methanol). Synthetic PSMα3 (Kelowna International Scientific Inc., Taiwan) was used as a size marker to indicate the location of PSMs on the SDS-PAGE gel. The total amounts of exoproteins in the culture medium were used as an internal control. The intensity of protein bands in each lane was quantified by using a densitometer. The amount of PSMs was normalized to the amount of exoproteins.

### Assessment of δ-Hemolytic Activity

Expression of δ-hemolysin was evaluated by cross-streaking test strains perpendicularly to *S. aureus* RN4220, which produces β-hemolysin, according to the method described previously (Herbert et al., 2010). β-Hemolysin enhances δ-hemolysin-mediated lysis of sheep red blood cells (Herbert et al., 2010). Therefore, δ-hemolytic activity can be visualized as a zone of enhanced hemolysis at the intersection of *S. aureus* RN4220 and the streaked test strain.

### Determination of Agr Expression in Spreading Colonies

Activation of the *agr* operon was determined according to a method described previously (Lin et al., 2016). In brief, *S. aureus* HG001(pPSM-*gfp*) and *S. aureus* M60(pPSM-*gfp*) were inoculated on TSA-0.4 agar and cultured at 37°C for 5 h. Colonies that formed on TSA-0.4 were observed under an upright confocal laser-scanning microscope (CLSM) (Leica, TCS-SP2). Green fluorescence expression by the colony indicated quorum-sensing activation.

### ATP Assay

Tryptic soy agar-0.4 plates were inoculated with 2 μl overnight bacterial culture and incubated at 37°C for 3 h. The cells on the surface of the TSA-0.4 plates were harvested, washed, suspended in PBS, and enumerated spectrophotometrically at OD_578_. The amount of intracellular ATP was measured by using a BacTiter-Glo Microbial Cell Viability Assay Kit (Promega, Madison, WI, United States). A 100-μl aliquot of cells was mixed with an equal volume of the assay reagent. The mixture was incubated at room temperature with shaking for 10 min. After incubation, the luminescence signal, which is proportional to the amount of ATP, was detected using a luminometer (Promega, GloMax Explorer GM-3510). ATP concentration was calculated based on an ATP standard curve that was prepared using adenosine 5′-triphosphate. The ATP level in the cells was normalized to the absorbance of the bacterial culture at 578 nm (*A*_578_).

### Measurement of Cytochrome Oxidase Activity

The enzyme activity was determined using cytochrome *c* oxidase assay kit (CYTOCOX1, Sigma–Aldrich, Inc., St. Louis, MO, United States) according to the manufacturer’s instructions. A 2-μl overnight bacterial culture was inoculated on TSA-0.4 plates. After incubation at 37°C for 3 h, the cells on the surface of the plates were harvested, washed, and enumerated spectrophotometrically at OD_578_. Bacterial suspensions were mixed with assay buffer and ferrocytochrome *c* substrate solution. The decrease in absorbance at 550 nm of ferrocytochrome *c* caused by its oxidation to ferricytochrome *c* was measured. Enzyme activity was then calculated and presented as Units/ml. Definition of one unit is to oxidize 1.0 μ mole of ferrocytochrome *c* per min at pH 7.0 and 25°C. The value of each sample was normalized to the absorbance of the bacterial culture at 578 nm (*A*_578_).

### RNA Isolation and Real-Time RT-PCR

Bacterial cells were suspended in 0.5 mg/ml lysostaphin (Sigma–Aldrich, Inc., St. Louis, MO, United States) and incubated at 37°C for 15 min. RNA was then isolated and purified by using TRIzol reagent (Invitrogen, Waltham, MA, United States) according to the manufacturer’s instructions. The transcripts of *qoxA* and *atpA* were reverse transcribed and amplified using the primers: qoxA-F (5′-CATTTGTATCCTGCACTTACTG) and qoxA-R (5′-CGTTGCTGCTTTAGCTATTC); atpA-F (5′-TGGTGTTCGACTGGTAATG) and atpA-R (5′-TTC GGTTCAGACCTTGAT), respectively. The *gyrB* mRNA, which was used as an internal control, was amplified using primers F1 (5′-ACGGATAACGGACGTGGTATCCCA) and R1 (5′-GCCACCGCCGAATTTACCACCA). RNA transcripts were quantified by real-time reverse transcription-PCR (RT-PCR; Bio-Rad). To monitor the specificity of RT-PCR, the PCR products were analyzed by melting-curve analysis. Experiment was performed at least three times and each sample was prepared in triplicate.

### Statistical Analysis

Data were analyzed by two-tailed Student’s *t*-test using GraphPad Prism software version 5.0 (La Jolla, CA, United States). A *p*-value of <0.05 was considered statistically significant. Data are presented as the mean ± SD.

## Results

### Involvement of the Heme Synthesis System in Colony Spreading

**FIGURE 1:**
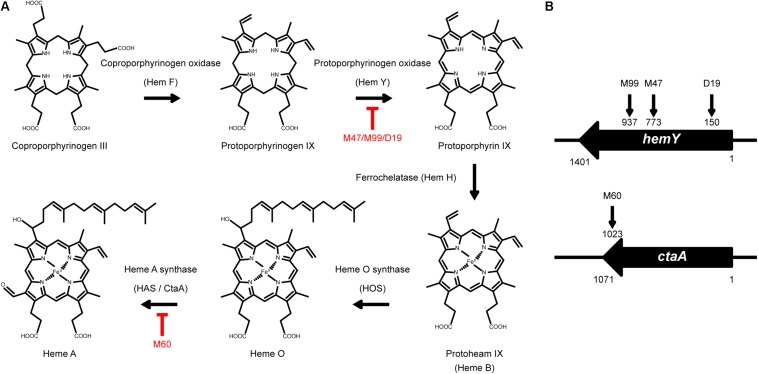
The heme synthesis pathway and transposon insertion mutations in non-spreading mutants. **(A)** Schematic illustration of the heme synthesis pathway and mutants that are defective in heme synthesis. **(B)** The vertical arrows on *hemY* and *ctaA* indicate the location of transposon insertions in M99, M47, D19, and M60.

**FIGURE 2:**
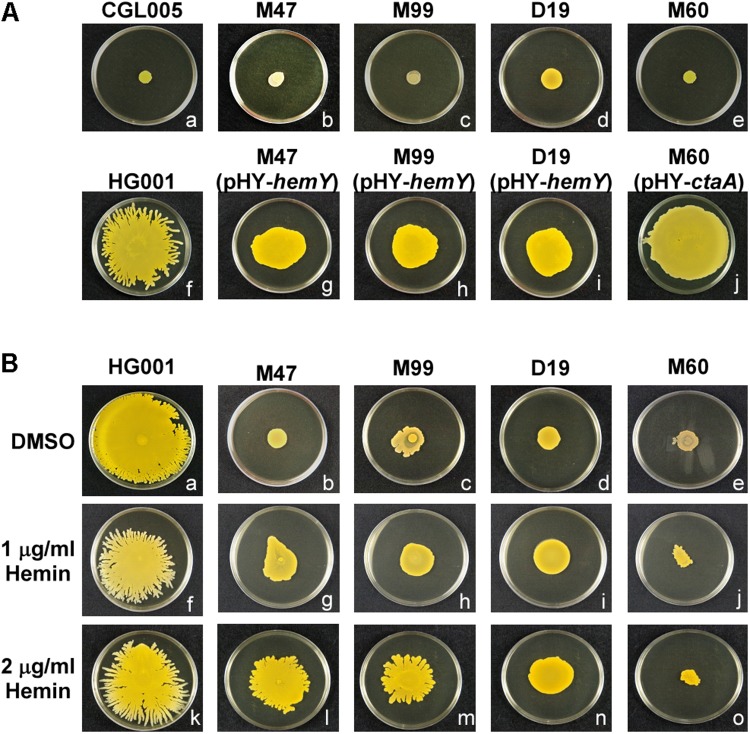
Morphology of spreading colonies formed by heme-deficient mutants. **(A)** *S. aureus* strains CGL005 **(a)**, M47 **(b)**, M99 **(c)**, D19 **(d)**, M60 **(e)**, HG001 **(f)**, M47 (pHY-*hemY*) **(g)**, M99 (pHY-*hemY*) **(h)**, D19 (pHY-*hemY*) **(i)**, and M60 (pHY-*ctaA*) **(j)** were inoculated on TSA-0.25 plates. After 24 h of incubation at 37°C, the morphology of spreading colonies was observed. Strain CGL005 is a non-spreading mutant. **(B)** TSA-0.25 plates containing 1 μg/ml **(f–j)** or 2 μg/ml **(k–o)** hemin were inoculated with *S. aureus* strains HG001 **(a,f,k)**, M47 **(b,g,l)**, M99 **(c,h,m)**, D19 **(d,i,n)**, and M60 **(e,j,o)**. After incubation for 24 h, morphology of spreading colonies was observed. Plates containing DMSO **(a–e)** were used as a solvent control. The pictures shown are representative of at least three independent experiments.

To identify the genes that are involved in colony spreading, *S. aureus* HG001 was mutagenized with a mariner-based transposon, *bursa aurealis* (Bae et al., 2004). Approximately 9000 colonies harboring transposon insertions were obtained. These colonies were subsequently screened for their ability to spread on TSA-0.25. Among the mutants that were defective in colony spreading, M253, M480, and CGL005 contained transposon insertions in *agrC* and *agrD* (Table 1), verifying the importance of the Agr quorum-sensing system in *S. aureus* spreading (Tsompanidou et al., 2011). Mutants M47, M99, and D19 had a transposon insertion in different locations in *hemY* (Figure 1B). *HemY* encodes protoporphyrinogen oxidase, which converts protoporphyrinogen IX into protoporphrin IX, a precursor of protoheam (heme B) (Panek and O’Brian, 2002; Figure 1A). Mutant M60 contained an insertion in *ctaA*, which encodes heme A synthase (Hederstedt, 2012). When these mutants were genetically complemented with a plasmid carrying *hemY* or *ctaA*, the colony spreading activities of the complemented mutants were restored (Figures 2Ag–j), indicating that *hemY* and *ctaA* are crucial to colony spreading.

We also incorporated hemin, a heme B derivative, into the TSA-0.25 plates to determine whether hemin addition rescued the spreading defect of the heme-deficient mutants. The results showed that adding 1 or 2 μg/ml hemin into the TSA-0.25 plates restored the spreading ability of the M47, M99, and D19 mutants (Figures 2Bf–i,k–n). However, hemin could not restore the spreading ability of M60, indicating that heme A, which is located downstream of the heme synthesis pathway, is also crucial for *S. aureus* colony spreading (Figures 1A, 2Bj,o).

### Mutations Affecting Heme Synthesis Impair Water Accumulation in Spreading Colonies

**FIGURE 3:**
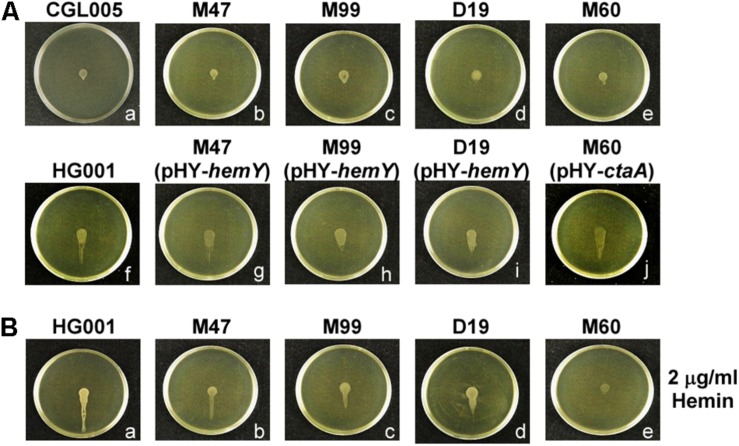
Water accumulation in spreading colonies. **(A)** TSA-0.4 plates were inoculated with overnight cultures of *S. aureus* strains CGL005 **(a)**, M47 **(b)**, M99 **(c)**, D19 **(d)**, M60 **(e)**, HG001 **(f)**, M47 (pHY-*hemY*) **(g)**, M99 (pHY-*hemY*) **(h)**, D19 (pHY-*hemY*) **(i)**, and M60 (pHY-*ctaA*) **(j)**. **(B)** TSA-0.4 plates containing 2 μg/ml hemin were inoculated with overnight cultures of *S. aureus* strains HG001 **(a)**, M47 **(b)**, M99 **(c)**, D19 **(d)**, and M60 **(e)**. Plates were tilted 30° and incubated for 3 h to observe the flow of water out of the colonies. Strain CGL005 is a non-spreading mutant. The pictures shown are representative of at least three independent experiments.

To determine whether heme deficiency affected water accumulation in spreading colonies, the plates were tilted at a 30° angle during culturing to observe the flow of accumulated water in the colonies with gravity. The tilted plate assay showed that after incubation for 3 h, water flowed out of the *S. aureus* HG001 colony toward gravity (Figure 3Af). However, only relatively small amount of water flowed out from M47, M60, M99, and D19 colonies (Figures 3Ab–e), indicating that mutations in heme synthesis genes affected water accumulation in the spreading colonies. Furthermore, water accumulation was restored in the spreading colonies of the complemented mutants (Figures 3Ag–j). Adding hemin to the medium also rescued the water accumulation phenotypes of the heme-deficient mutants M47, M99, and D19 (Figures 3Bb–d) but not that of M60 (Figure 3Be). These results verified that *hemY* and *ctaA* are critical not only for colony spreading but also for water accumulation.

### Activation of Quorum Sensing in Spreading Colonies of Heme-Deficient Mutants

**FIGURE 4:**
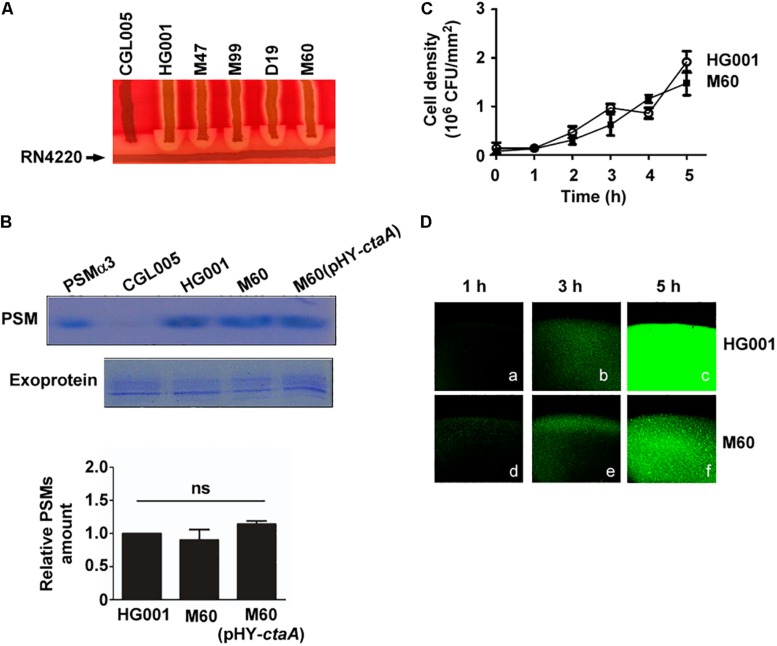
Agr activity in heme-deficient mutants. **(A)** δ-Hemolysin activities of *S. aureus* strains CGL005, HG001, M47, M99, D19, and M60 were determined by cross-streaking the strains perpendicularly to *S. aureus* RN4220 on blood agar plates. The picture shown is representative of at least three independent experiments. **(B)** PSMs and exoproteins were isolated from the media of *S. aureus* HG001, M60, and M60 (pHY-*ctaA*) cultures. PSMs were extracted with butanol. The exoproteins in the culture medium were isolated using Amicon Ultra-4 centrifugal filters. Both PSMs and exoproteins were analyzed by SDS-PAGE followed by Coomassie blue staining **(Top)**. Synthetic PSMα3 was used as a size marker to indicate the location of PSMs on the SDS-PAGE gel. Strain CGL005, which is an Agr deficiency mutant and does not produce PSMs, was used as a control. Exoproteins in culture medium were used as loading control. The intensity of protein bands in each lane was quantified by using a densitometer. The amount of PSMs was normalized to the amount of exoproteins. The relative amount of PSMs from *S. aureus* HG001 was set at 1 to calculate the relative amount of PSMs from strains M60 and M60 (pHY-*ctaA*) **(Bottom)**. The gel shown is representative of three independent experiments. Data are presented as the mean of three independent experiments. Error bars denote standard deviations. ns indicates no significant difference. **(C)** TSA-0.4 plates were inoculated with *S. aureus* HG001 and M60 and incubated for 5 h. The cell number in a spreading colony was determined by CFU enumeration. Cell density (CFU/mm^2^) was then calculated. Data are presented as the mean of three independent experiments. Error bars denote standard deviations. **(D)** After 1, 3, or 5 h of incubation on TSA-0.4 plates, images of spreading colonies formed by *S. aureus* HG001 (pPSM-*gfp*) **(a–c)** and M60 (pPSM-*gfp*) **(d–f)** were acquired under a CLSM.

It is well known that, in *S. aureus*, the cell density-dependent Agr quorum-sensing system regulates hemolysins and PSMs synthesis (Queck et al., 2008) and is required for colony spreading (Tsompanidou et al., 2011). Our previous study showed that an increased bacterial density within a spreading colony triggers the Agr quorum-sensing response to transcribe *psm* genes and produce PSMs, which facilitate colony spreading (Lin et al., 2016). To examine whether defects in heme synthesis affected the activity of the Agr system, resulting in the reduction of spreading, we analyzed Agr functions based on the production of δ-hemolysin and PSMs. Since δ-hemolysin-mediated lysis of sheep red blood cells is enhanced by β-hemolysin, which is produced by *S. aureus* RN4220 (Herbert et al., 2010), therefore, δ-hemolytic activity can be visualized as a zone of enhanced hemolysis at the intersection of *S. aureus* RN4220 and the streaked test strains. δ-Hemolysin assay showed an enhanced hemolytic area at the intersection of streaks of the heme-deficient mutants M47, M99, D19, and M60 and strain RN4220 after 24 h of incubation, indicating that the mutants produced δ-hemolysin (Figure 4A). To further evaluate Agr functions, we examined PSMs production by extracting PSMs from the media of *S. aureus* HG001, M60, and M60 (pHY-*ctaA*) cultures. PSMs are small peptides that are secreted into the culture medium. As antibodies monitoring exoproteins are unavailable, we used the total amount of exoproteins as an internal control to correct the relative amount of PSMs in each sample. The results demonstrated that there was no significant difference in the amount of PSMs produced by the three strains (Figure 4B), indicating that heme deficiency does not influence PSMs expression. Our previous study revealed that quorum-sensing occurs in spreading colonies of *S. aureus* HG001 after 3 h of culturing (Lin et al., 2016). The current study found that the cell density of the colony formed by the non-spreading mutant M60 was similar to that of *S. aureus* HG001 during the first 5 h of culture (Figure 4C). Furthermore, a reporter plasmid, pPSM-*gfp*, in which the promoter of the *psm*α operon that is controlled by Agr quorum-sensing, activates the transcription of a *gfp* reporter gene, was transformed into *S. aureus* HG001 and the M60 mutant. We found that under a CLSM, the cells in the colony of *S. aureus* HG001 did not exhibit much green fluorescence before 3 h culturing (Figures 4Da,b). However, strong green fluorescent signals were detected after 5 h incubation (Figure 4Dc). The similar results were observed in the colony of M60 mutant (Figures 4Dd–f). The CLSM analysis revealed that a quorum-sensing response occurred in the spreading colony of the *S. aureus* HG001 and M60 mutant (Figure 4D). Overall, these results clarified that the inability of the heme-deficient mutants to spread is not due to Agr dysfunction.

### Heme Deficiency Impairs Energy Production and Colony Spreading

**FIGURE 5:**
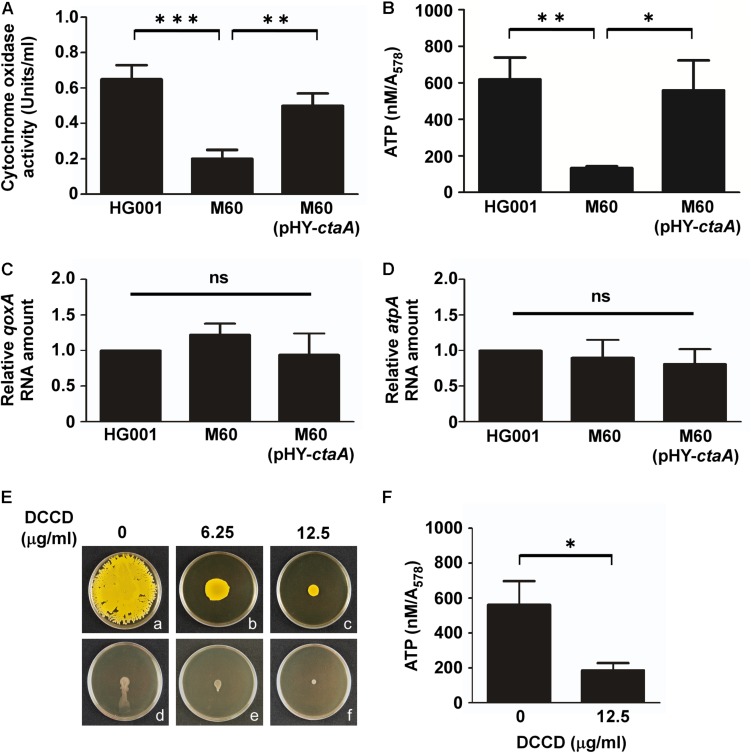
Mutation of *ctaA* and inhibition of ATPase decrease ATP production and colony spreading. After incubation on plates for 3 h, the cells in colonies of *S. aureus* HG001, M60, and M60 (pHY*-ctaA*) were harvested. The activity of cytochrome oxidase was determined using an enzyme assay kit **(A)**. The concentrations of intracellular ATP were determined and normalized to the absorbance of the bacterial cultures at 578 nm (*A*_578_) **(B)**. Total RNA was isolated from *S. aureus* HG001, M60, and M60 (pHY-*ctaA*). Transcriptions of *qoxA* **(C)** and *atpA* **(D)** were determined by quantitative RT-PCR. The amount of *qoxA* and *atpA* transcripts was normalized to the amount of *gyrB* RNA. The RNA amounts of *qoxA* and *atpA* in *S. aureus* HG001 were set at 1 to calculate the relative amounts of *qoxA* and *atpA* in strains M60 and M60 (pHY-*ctaA*). **(E)** *S. aureus* HG001 was inoculated on DCCD-containing TSA-0.25 **(a–c)** or TSA-0.4 **(d–f)** plates. After 24 h of incubation at 37°C, the morphology of spreading colonies on TSA-0.25 plates was observed **(a–c)**. TSA-0.4 plates containing DCCD were tilted 30° and incubated for 3 h to observe the flow of water out of the colonies **(d–f)**. **(F)** The cells on DCCD-containing TSA-0.4 plates were harvested for determination of the ATP concentrations. The pictures shown in part **(E)** are representative of at least three independent experiments. Data are presented as the mean of three independent experiments. Error bars denote standard deviations. Significant differences are denoted as ^∗^*p* < 0.05, ^∗∗^*p* < 0.01, and ^∗∗∗^*p* < 0.005. ns indicates no significant difference.

*Staphylococcus aureus* can utilize aerobic respiration to generate energy. Aerobic respiration depends on enzymes such as heme-containing cytochrome oxidases to catalyze a series of electron transport chain reactions, which drive proton translocation across the cytoplasmic membrane and produce ATP via the F_0_F_1_-ATPase (Mobius et al., 2010; Zeden et al., 2018). Therefore, we proposed that mutations in the heme synthesis pathway may impair cytochrome oxidase activity and decrease energy production. To test this hypothesis, cytochrome oxidase activity assays and ATP assays were conducted. The results showed that the activity of cytochrome oxidases in *S. aureus* HG001 was 0.65 unit/ml (Figure 5A). In the heme-deficient mutant M60, the activity of cytochrome oxidases decreased to 0.2 unit/ml (Figure 5A). However, the activity of cytochrome oxidases increased to 0.5 unit/ml when pHY-*ctaA* was introduced into M60 (Figure 5A). Similar results were obtained for detection of ATP production (Figure 5B). The results revealed that lack of heme A diminished the activity of cytochrome oxidases and led to decrease ATP production. Furthermore, heme A deficiency did not affect the transcription of *qoxA* and *atpA*, which encode cytochrome oxidase subunit and ATPase subunit, respectively (Figures 5C,D). In addition, an inhibitor of ATPase, DCCD (Joung et al., 2016), was used to clarify whether the requirement of energy is crucial to water accumulation and colony spreading. The results showed that adding DCCD into culture medium not only reduced ATP production (Figure 5F) but also decreased water accumulation (Figures 5Ed–f) and colony spreading (Figures 5Ea–c). These results revealed that heme deficiency impaired ATP production and resulted in decreased water accumulation and colony spreading.

## Discussion

Heme is the prosthetic group of cytochrome oxidases, which are important for electron transport and energy production (Mobius et al., 2010; Hederstedt, 2012). Previous studies have shown that electron-transport-defective mutants have characteristics similar to those of small cell variants (SCVs), which exhibit reduced colony size and slow growth (von Eiff et al., 1997; Hammer et al., 2013). Despite that our heme-deficient mutants exhibited phenotypes typical of SCVs, such as slow growth and decreased colony size, the cell densities of the colonies formed by the mutants reached the threshold required to trigger the quorum-sensing response to initiate colony spreading (Figures 4C,D). Although both strains HG001 and M60 reached a comparable cell density to trigger the quorum-sensing response (Figures 4C,D), the total number of cells in the colony of the M60 mutant is less than that of the strains HG001 because of the decreased colony size of the M60 mutant. Therefore, under a CLSM, the fluorescence intensity of the strains HG001 is stronger than that of the M60 mutant (Figure 4D). Furthermore, defects in heme synthesis had little effect on the expression of PSMs (Figure 4B). These results indicate that the non-spreading phenotype of the heme-deficient mutants is not attributable to slow growth or Agr dysfunction. However, the colony spreading assay and tiled plate assay showed that mutations in heme synthesis genes affected water accumulation and colony spreading (Figures 2, 3). We also used another heme deficiency mutant *S. aureus* NCTC8325-4Δ*hemB*, which also exhibited typical phenotypes of SCVs, to clarify the importance of heme synthesis to water accumulation and colony spreading (Supplementary Figure S2).

*Staphylococcus aureus* is able to spread on a moist semisolid surface (Kaito and Sekimizu, 2007). Our earlier study also showed that increasing the agarose concentration decreases colony spreading (Lin et al., 2016). However, increasing the volume of culture medium in the plate can rescue *S. aureus* spreading on plates containing a high concentration of agarose (Lin et al., 2016). These results indicate that the amount of water in the medium influences the ability of *S. aureus* to spread. Additionally, there is overwhelming evidence supporting the idea that water is recruited from the culture medium by a bacterial swarm (Wu and Berg, 2012; Ping et al., 2014; Ke et al., 2015; Yang et al., 2017). Work by Wu and Berg (2012) showed that a water reservoir traveling with a swarm fuels the spreading of *E. coli*. *B. subtilis* swarm in water, and the water surface tension modulates the swarming mechanics (Ke et al., 2015). A recent study on the swarming motility of *P. aeruginosa* demonstrated that their ability to draw water from the culture medium is the essential factor determining whether this bacteria can spread over the agar surface (Yang et al., 2017). Similarly, our previous study showed that water accumulation in spreading colonies to elicit water surface tension and is crucial for facilitating colony spreading of *S. aureus* (Lin et al., 2016). However, the underlying mechanism by which *S. aureus* extracts water from the medium is still unknown.

Based on the screening of a transposon mutant library, we found that mutations in genes involved in heme synthesis impaired the spreading of *S. aureus* (Table 1). When the heme-deficient mutants were cultured on tilted plates, the colonies formed by the mutants had less water flowing out (Figure 3A), suggesting that heme synthesis affects water accumulation during colony spreading of *S. aureus*. Since hemes are involved in energy production, we hypothesize that *S. aureus* utilizes an energy-dependent mechanism to extract water from the culture medium. Water accumulation in a colony increases the surface tension to restrict colony expansion. The cells grow, and the cell density in the colony increases, which triggers the quorum-sensing response to activate expression of PSMs. As water is continuously extracted from the medium, PSMs weaken the water surface tension, allowing water to flow. *S. aureus* is then carried by the flowing water across the plate surface.

Although many studies strongly suggest that water recruitment is crucial for bacterial swarming and colony spreading, the exact molecule to extract water is still unknown. In *E. coli*, it has been suggested that LPS may serve as the osmolyte to extract water from agar and facilitates its swarming (Ping et al., 2014). In *S. aureus*, mutations in the *tagO* gene, which encodes the first enzyme TagO of the WTAs synthesis pathway, impair the spreading ability of *S. aureus* (Kaito and Sekimizu, 2007), indicating WTAs are required for colony spreading of *S. aureus*. Both LPS and WTAs are glycopolymers that attached to the cell surface of bacteria. Therefore, we posit that WTAs are likely as an osmolyte and involved in water extraction during colony spreading of *S. aureus*. To test the hypothesis, *S. aureus* HG001 was cultured on plates that contained tunicamycin, an inhibitor of TagO (Campbell et al., 2011). The results showed that tunicamycin inhibited colony spreading of *S. aureus* in a dose dependent manner (Supplementary Figure S1A). Furthermore, inhibition of TagO by tunicamycin (Supplementary Figure S1A) or deletion of *tagO* in *S. aureus* strain SA113 (SA113Δ*tagO*) (Supplementary Figure S1B) decreased water accumulation in spreading colony, indicating that WTAs are required for water extraction during colony spreading of *S. aureus*. WTAs are synthesized intracellularly and exported to the cell surface by an energy-dependent ABC (ATP-binding cassette) transporter (Winstel et al., 2014), indicating that energy is required to facilitate translocation of WTAs across membrane. Accordingly, lack of heme in heme deficiency mutants results in reduction of ATP production and may decrease the amount of WTAs attached to cell surface, by which diminish the water extraction and disable colony spreading. However, the mechanisms need to be further investigated.

Although the ability of *S. aureus* to move across a soft solid surface is well known, colony spreading of *S. aureus* is currently defined as a form of passive motility (Pollitt and Diggle, 2017). A recent study showed that *S. aureus* forms comet structures at the tips of spreading colony dendrites that facilitate colonies extension on soft agar (Pollitt et al., 2015). This comet-type movement exhibits the characteristics of active motility (Pollitt et al., 2015). The current study finds that heme is required for both energy production and colony spreading. Additionally, the results indicate that energy consumption is involved in colony spreading and imply that colony spreading is a form of energy-dependent active motility. Furthermore, both colony spreading and comet motility of *S. aureus* require Agr-induced expression of PSM-type biosurfactants (Pollitt et al., 2015; Kizaki et al., 2016). Biosurfactants are also required for the swarming motility of *B. subtilis*, *Serratia marcescens*, and *Pseudomonas aeruginosa* (Kearns, 2010), suggesting that *S. aureus* shares a common mechanism with these actively motile bacteria to move on agar surfaces.

In nature, the ability to move rapidly gives bacteria advantages to obtain nutrient and food. Moreover, the significance of motility in bacterial pathogenesis is well documented (Jones et al., 2004). It is known that the motility of *P. mirabilis*, *E. coli*, *Vibrio cholera*, and *S. marcescens* is closely associated with their pathogenesis (Jones et al., 2004; Syed et al., 2009; Shanks et al., 2013; Garcia-Heredia et al., 2016). An earlier study showed that *S. aureus* is capable of spreading on fresh pork meat, showing that this organism is able to spread on soft tissues (Tsompanidou et al., 2013). Previous studies also demonstrated that most invasive *S. aureus* clinical strains are able to spread, indicating that spreading is a common characteristic shared by strains that cause invasive infections in human (Kaito et al., 2008). The results of this study provide new insights into the movement mechanisms of bacteria that lack a motor apparatus and elucidate how *S. aureus* spreads, which is important for understanding how this pathogen moves in human tissues to cause disease.

## Data Availability Statement

The raw data supporting the conclusions of this article will be made available by the authors, without undue reservation, to any qualified researcher.

## Author Contributions

M-HL and C-CL conceived and designed the study, performed the experiments, analyzed the data, and wrote the manuscript.

## Conflict of Interest

The authors declare that the research was conducted in the absence of any commercial or financial relationships that could be construed as a potential conflict of interest.
