# Supplementary material for: Involvement of Heme in Colony Spreading of Staphylococcus aureus
Source: Front Microbiol. 2020 Feb 11;11:170. doi: 10.3389/fmicb.2020.00170 (PMC7026375; doi:10.3389/fmicb.2020.00170)
Supplement: Supplementary file 1 [file Data_Sheet_1.PDF]

Supplementary Material

Supplementary Figure S1

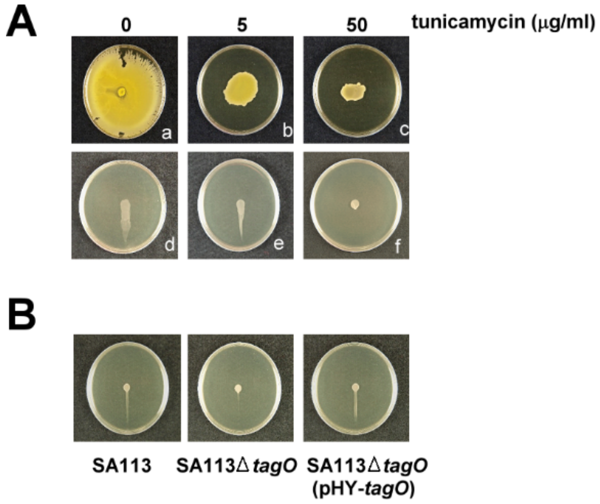

Supplementary Figure S1. Involvement of wall teichoic acid in water accumulation and colony spreading of *S. aureus*. **(A)** TSA-0.25 (a-c) or TSA-0.4 (d-f) plates containing 0 μg/ml (a, d), 5 μg/ml (b, e) or 50 μg/ml (c, f) tunicamycin were inoculated with overnight cultures of *S. aureus* HG001. After incubation for 24 h, morphology of spreading colonies was observed (a-c). To observe the flow of water out of the colonies, TSA-0.4 plates were tilted 30° and incubated for 5 h (d-f). **(B)** Overnight cultures of *S. aureus* strains SA113, SA113Δ*tagO* and SA113Δ*tagO*(pHY-*tagO*) were inoculated on TSA-0.4 plates. Plates were tilted 30° and incubated for 5 h to observe the flow of water out of the colonies. The pictures shown are representative of at least three independent experiments.

Supplementary Figure S2

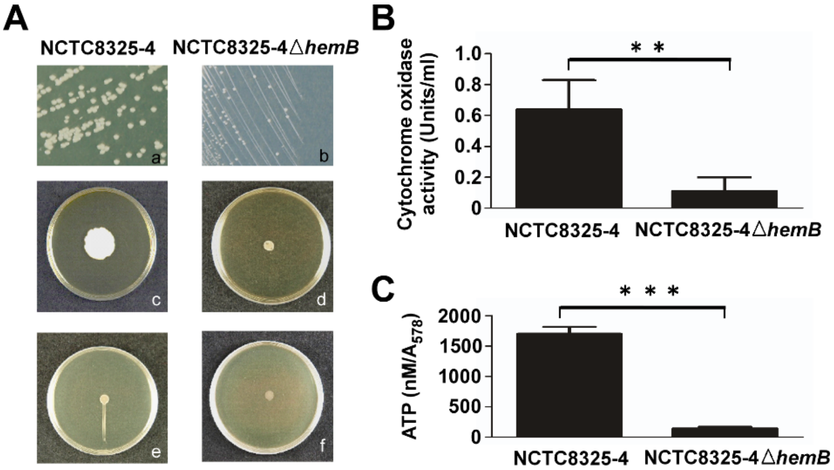

Supplementary Figure S2. Mutation of *hemB* decreases cytochrome oxidases activity, intracellular ATP levels and colony spreading. **(A)** *S. aureus* NCTC8325-4 and NCTC8325-4Δ*hemB* were inoculated on TSA (a, b), TSA-0.25 (c, d) and TSA-0.4 (e, f). After 24 h of incubation at 37°C, colony morphology (a, b) and colony spreading (c, d) were observed. TSA-0.4 plates were tilted 30° and incubated for 5 h to observe the flow of water out of the colonies (e, f). **(B)** The activity of cytochrome oxidase were determined using an enzyme assay kit. **(C)** The concentrations of intracellular ATP were determined using an ATP assay kit and normalized to the absorbance of the bacterial cultures at A_{578}. The pictures in (A) are representative of at least three independent experiments. Data are presented as the mean of three independent experiments. Error bars denote standard deviations. Significant differences are denoted as *$p < 0.05$, **$p < 0.01$ and ***$p < 0.005$.
